# Does tamsulosin facilitate expulsion of distal ureteric calculus following lithotripsy?

**Published:** 2008

**Authors:** Gaurav Gupta, Karthikeyan Aswathaman, Nitin S. Kekre

**Affiliations:** Department of Urology, Christian Medical College, Vellore - 632 004, Tamil Nadu, India. E-mail: uro2@cmcvellore.ac.in

## SUMMARY

The authors in this randomized control trial have investigated whether addition of tamsulosin can facilitate clearance of distal ureteric stone following extracorporeal shock wave lithotripsy (ESWL).[1] Sixty-one consecutive patients (38 men and 23 women) with single distal radiopaque ureteral calculus of 6 mm or more in diameter were enrolled. To reach a 0.8 power in the study, it was estimated that for a significance level (α) of 0.05 and effect size of 0.35, a sample size of 64 patients was required. The differences between the two groups were tested using the χ^2^-test and the Mann-Whitney *U*-test. The analysis was done on intension to treat basis. After ESWL they were randomized into two groups. Nonsteroidal antiinflammatory drug (diclofenac 50 mg) was given to both the groups upon demand. In addition, all the patients in group B (*n* = 30) received once a day tamsulosin (0.4 mg). Follow-up visits were performed at 1, 2, 3, and 4 weeks after ESWL, evaluation included a plain X-ray KUB and an ultrasound. Efficacy of tamsulosin was evaluated in terms of success rate, stone-free rate, time to expulsion of fragments, and use of diclofenac. Two patients from the tamsulosin group experienced dizziness and one withdrew from the study. The success rate was 58 and 67% for the control and the tamsulosin group, respectively. The stone-free rate was 52 and 63%, respectively. The mean time to expulsion of the fragments was 13.22 days in group A and 12.95 days in group B. The success rate, stone free rate, and time to expulsion of fragments were not statistically significant (*P* > 0.05). The mean diclofenac dose was 119 mg in group A and 57 mg in group B which was statistically significant (*P* = 0.02). Despite the relatively small number of patients, authors clearly showed that tamsulosin following ESWL in this specific subgroup of patients did not result in improved success and stone-free rate. In contrast, a significantly reduced need for analgesics was found.

## COMMENTS

Alpha-1 adrenoceptors have been identified in human ureter. It has been hypothesized that α-blockers inhibits peristaltic activity and relaxes the basal tone of the ureter thereby facilitating expulsion of the ureteric calculus. Majority of the randomised trials have shown that tamsulosin is effective in expulsion of ureteric calculus. Unfortunately, there is no uniformity in the inclusion criteria or in the regimens in these trials. Furthermore, different agents, doses, duration of treatment, adjunctive drugs, variability in calculus size, and location make comparison between trials difficult.[2] In a meta-analysis of nine randomized control trials, alpha blockers and calcium channel blockers were found to be effective.[3] Contrary to the evidence, the authors in the present study did not find any statistical significant difference between the two groups. The mean stone size was 9 mm. Tamsulosin has been shown to improve the clearance of large renal stones after ESWL compared to smaller stones.[4] Küpeli *et al*.[5] observed that tamsulosin was effective in clearance of lower ureteric calculus following lithotripsy. The stone free rates in those who received and did not receive tamsulosin were 71 and 33%, respectively. In a randomized control trial with tamsulosin on ureteric steinstrasse, tamsulosin was not found to be effective in clearance of steinstrasse.[6] Differences in the reported efficacy of tamsulosin may be related to the type of lithotriptor, size, composition, and degree of impaction of the calculus. Present study is not a double blind randomized controlled trial, so the conclusion that “adjunctive administration of tamsulosin can reduce the total analgesic consumption after an ESWL” should be interpreted with caution. Multicentric randomized control trials are required to prove the efficacy of tamsulosin following lithotripsy, till then its use is clinician's choice.
